# The reverse genetics applied to fish RNA viruses

**DOI:** 10.1186/1297-9716-42-12

**Published:** 2011-01-24

**Authors:** Stéphane Biacchesi

**Affiliations:** 1 Unité de Virologie et Immunologie Moléculaires, INRA, CRJ, 78352 Jouy-en-Josas, France

## Abstract

Aquaculture has expanded rapidly to become a major economic and food-producing sector worldwide these last 30 years. In parallel, viral diseases have emerged and rapidly spread from farm to farm causing enormous economic losses. The most problematic viruses encountered in the field are mainly, but not exclusively, RNA viruses belonging to the *Novirhabdovirus*, *Aquabirnavirus*, *Alphavirus *and *Betanodavirus *genera. The recent establishment of reverse genetics systems to recover infectious fish RNA viruses entirely from cDNA has made possible to genetically manipulate the viral genome. These systems have provided powerful tools to study all aspects of the virus biology and virus-host interactions but also gave the opportunity to use these viruses as live vaccines or as gene vectors. This review provides an overview on the recent breakthroughs achieved by using these reverse genetics systems in terms of viral protein function, virulence and host-specificity factor, vaccine development and vector design.

## Introduction

Fish farmers are faced worldwide to viral infections which destroy each year a significant part of the production and thus induce important economic losses [1,2]. In addition to direct losses and delay in the aquaculture expansion, viral diseases also cause additional impacts including shortage of food and jobs as well as social and environmental costs. Major viral diseases are mainly due to two antigenetically distinct *Novirhabdoviruses *which can coexist in the same fish farms: the viral hemorrhagic septicemia virus (VHSV) and the infectious hematopoietic necrosis virus (IHNV). Other viruses have also a serious impact in the field such as the infectious pancreatic necrosis virus (IPNV), an *Aquabirnavirus *infecting for instance salmonid and viruses belonging to the *Betanodavirus *genus which cause serious diseases in several marine fish species. The genome of these viruses consists of either a negative-sense single-stranded RNA molecule for the *Novirhabdoviruses *of about 11 kilobases (kb) [3,4], two segments of double-stranded RNA (A and B) of about 2.8 and 3.1 kilobase pairs (kbp) each for the *Aquabirnavirus *[5,6] or two positive-sense single-stranded RNA (RNA1 and RNA2) of about 1.4 and 3.1 kb each for the *Betanodaviruses *[7,8].

These viral diseases are the most frequent but due to the expansion of the salmonid aquaculture and the development of new fish species production, new diseases have emerged in the recent years [2]. One of the most striking examples is undoubtedly the emergence of the sleeping disease in farmed rainbow trout (*Oncorhynchus mykiss*) and the pancreas disease in marine-reared Atlantic salmon (*Salmo salar*). These diseases are known since the 80s and have now spread in most of the European countries and in North America. For instance, the sleeping disease may affect 30 to 40% of the French fish farms. Viruses responsible for these diseases, the sleeping disease virus (SDV) and the salmon pancreas disease virus (SPDV) belong to the *Alphavirus *genus, with a genome which consists of a positive-sense single-stranded RNA molecule of about 12 kb [9,10].

Since many years, goals of different laboratories around the world have been mainly but not exclusively devoted to the development of new vaccine approaches based in part on live attenuated viruses to prevent viral diseases in aquaculture [11,12] and the determination of potential genetic factors that could explain the differences of virulence existing between virus strains and their host range restriction. For that, a way allowing the manipulation of the viral genome is required. A major step in engineering a viral RNA genome is the ability to recover live virus from a DNA copy of the RNA genome: this is called "reverse genetics". First success in reverse genetics for a positive-stranded RNA virus, the poliovirus (*Picornaviridae *family), was in 1981 [13]. Thirteen years later, a reverse genetics system was established for the recovery of a nonsegmented negative-stranded RNA virus, the rabies virus (RV) [14]. The ability to manipulate a cDNA intermediate, exact copy of the viral RNA genome, opens the possibility of deleting genes for studying their function, introducing targeted mutations to determine potential genetic factors of virulence or inserting heterologous genes of interest and using these recombinant viruses as gene vectors.

Reverse genetics systems have been now established for several fish RNA viruses such as the *Novirhabdoviruses *IHNV and VHSV [15-18], the *Alphavirus *SDV [19], the *Aquabirnavirus *IPNV [20] and also for several *Betanodavirus *species such as for the striped jack nervous necrosis virus (SJNNV) [21]. A large number of various recombinant viruses have been generated by different groups. Some of them are currently being evaluated as live vaccines in field trials and some others are being used as gene vectors in fish. Some examples of such recombinant viruses will be described and the advances in terms of vaccine development, virulence and host-specificity factor mapping and vector design will be discussed.

## Reverse genetics systems: description and technical tricks

### Alphavirus

Salmonid *Alphaviruses *are recognized as serious pathogens of farmed Atlantic salmon and rainbow trout in Europe [9]. Sleeping disease (SD) is an infectious disease of rainbow trout reared in fresh water while salmon pancreas disease (SPD) has been recorded in farmed Atlantic salmon. The aetiological agents responsible for these diseases, namely SDV and SPDV, respectively, are closely related viruses that belong to the *Alphavirus *genus of the *Togaviridae *family [10,22]. Sindbis virus (SINV), one the most studied virus among the *Alphavirus *genus, is transmitted by mosquitoes, and its alternate vertebrate host is usually a bird or a mammal. Like all Alphaviruses, SDV and SPDV virions are spherical (70 nm in diameter), with a lipid envelop containing heterodimeric glycoprotein spikes composed of two virus glycoproteins (E1 and E2). Some alphaviruses may contain a third envelop protein (E3). The envelope is tightly organized around an icosahedral nucleocapsid which contains the genomic RNA. The viral genome consists of a positive-sense single-stranded RNA molecule of about 12 kb in length, which is capped at the 5'-terminus and polyadenylated at the 3'-terminus, and contains two open reading frames (ORF). The 5' two-thirds of the genome encode four non-structural proteins (nsP1 to nsP4) that are translated directly from the genomic RNA as a polyprotein precursor, which undergoes subsequent proteolytic cleavage. The four non-structural proteins are involved in genome replication and the synthesis of a subgenomic RNA from the negative-strand copy of the genome that corresponds to the 3' third of the viral genome and encodes the second ORF. This second ORF is translated as a polyprotein, which is processed to produce the viral structural proteins: the capsid, the glycoproteins E3, E2, E1 and the 6K protein. In contrast to the other Alphaviruses, an arthropod-independent transmission has been demonstrated for salmonid Alphaviruses by cohabitation experiments [9].

**Figure 1:**
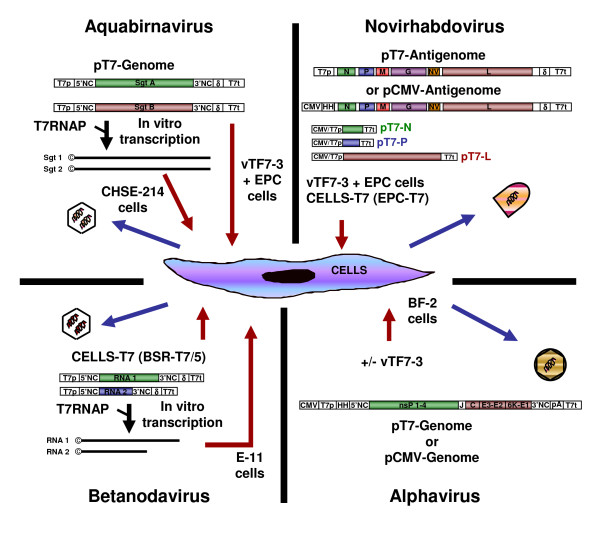
**Systems of reverse genetics for fish RNA virus generation entirely from cloned cDNA**. Methods used for the recovery from plasmid cDNA of fish RNA viruses, belonging to the *Aquabirnavirus*, *Novirhabdovirus*, *Betanodavirus *and *Alphavirus *genera, are presented. A schematic representation of each plasmid cDNA encoding viral replicative complex proteins or containing a cDNA copy of full-length viral genome, antigenome or genome segments is drawn. Plasmid DNA or in vitro transcribed RNA were transfected (red arrows) in either untreated fish permissive cells (CHSE-214, E-11 and BF-2 cells) or permissive cells constitutively expressing the T7RNAP (CELLS-T7: fish EPC-T7 cells or Baby Hamster Kidney derived BSR-T7/5 cells) or previously infected with a recombinant vaccinia virus (vTF7-3) encoding the T7RNAP (EPC and BF-2 cells). After the initiation of an infectious cycle, recombinant virus can be harvested (blue arrows). For further details, see section 2. Abbreviations are: T7p, T7 promoter; CMV, cytomegalovirus promoter; HH, hammerhead ribozyme sequence; NC, non-coding region; Sgt, viral genome segment; pA, polyadenylation signal; δ, antigenomic sequence of a ribozyme derived from the hepatitis delta virus; T7t, T7 terminator; **©**, cap structure; T7RNAP, T7 RNA polymerase.

Based on the observation that genomic RNA from most of the positive-stranded RNA viruses could serve as messenger RNA (mRNA) and initiate infection upon transfection in a permissive cell line, the recovery of infectious virus from complementary DNA (cDNA) was first described for poliovirus by Racaniello and Baltimore, almost 30 years ago [13]. The first infectious cDNA for a member of the *Alphavirus *genus was established for SINV few years later by Rice et al. [23]. The recovery of recombinant alphavirus from cDNA was typically based on the transfection into cells of synthetic capped RNA transcripts generated by in vitro transcription from SP6- or T7-driven full-length viral cDNA constructs. Although less efficient than transfection of in vitro-synthesized RNA, transfection of viral genome cDNA copy under the control of a RNA polymerase II promoter (the cytomegalovirus (CMV) immediate early promoter) was also described (for review [24]). Similar approaches for SDV failed to produce infectious recombinant virus [19,25]. The key to recover SDV was found by fusing a self-cleaving hammerhead ribozyme sequence at the 5'-end of the cDNA copy of SDV full-length genome [19]. Indeed, a precise 5'-end of SDV RNA genome appears to be crucial to initiate an infectious cycle. This cDNA construct was then cloned under the control of a T7 RNA polymerase (T7RNAP) promoter (Figure 1) and recombinant SDV was recovered upon transfection of this cDNA plasmid into fish cells (bluegill fry BF-2 cells) previously infected by a recombinant vaccinia virus (vTF7-3; [26]) expressing the T7RNAP. Because of the temperature requirement of each virus (37°C and 10°C for vTF7-3 and SDV, respectively), cells were first incubated at least 7 h at 37°C to allow their infection by the vTF7-3 and the expression of enough functional T7RNAP before being shifted at 10°C following the protocol previously described for fish rhabdovirus minigenome rescue ([27] and section 2.4). This system was further simplified by the exchange of the T7RNAP promoter by that of the CMV avoiding the restrictive use of the vTF7-3 [19]. A similar cDNA construct was recently used with success for the expression of a salmon alphavirus subtype 3 replicon in fish cells [28].

### Aquabirnavirus

Infectious pancreatic necrosis (IPN) is a serious disease of salmonid and a number of other fish species that can lead to high mortality (70%) in first feeding fry and fingerlings of most salmonids. Furthermore, the aetiological agent responsible for the disease (IPNV) can be vertically transmitted via the egg and broodstock carrier fish facilitating its rapid spread to new geographic area and its expansion to other farmed fish species [29]. IPNV is the type species of the *Aquabirnavirus *genus of the *Birnaviridae *family. Infectious bursal disease virus (IBDV), another member of *Birnaviridae *family that has been studied in the greatest detail is the causative agent of highly immunosuppressive disease in young chickens and belongs to another genus, *Avibirnavirus *[5]. IPNV is a non-enveloped icosahedral capsid virus (60 nm in diameter) that contains two segments (A and B) of double-stranded RNA [30]. Segment A (about 3.1 kbp) contains two ORF. ORF2 encodes a 106-kDa precursor protein, which is autoproteolytically cleaved by the viral encoded protease (VP4 or NS for non structural) to generate the viral capsid proteins VP2 and VP3. Segment A also encodes an additional non structural protein VP5 from the small ORF1, which precedes and partly overlaps the large ORF2. Segment B (about 2.8 kbp) encodes a 94-kDa protein, called VP1, which is the viral associated RNA-dependant RNA polymerase (RdRp).

The first description of segmented double-stranded RNA virus recovery entirely from cDNA was given by Mundt and Vakharia for IBDV [31]. Few years later, Yao and Vakharia used a similar approach to recover IPNV and thus reporting the first reverse genetics system for a RNA virus infecting an aquatic species [20]. In both cases, synthetic positive-sense RNA transcripts of segments A and B were transcribed in vitro from linearized full-length cDNA plasmids with the T7RNAP in the presence of a synthetic cap analogue and used to transfect permissive cells (chinook salmon CHSE-214 cells) (Figure 1). IPNV recovery was also achieved upon transfection of vTF7-3-infected fish cells (fathead minnow EPC (*epithelioma papulosum cyprinid*) cells) with segment A and B cDNA copies (Biacchesi S., unpublished data). Segments A and B were cloned under the control of a T7RNAP promoter and upstream the antigenomic sequence of a self-cleaving ribozyme derived from the hepatitis delta virus (δ) allowing the expression by the T7RNAP of RNA molecules displaying a precise 3'-end without any additional nucleotide [32].

### Betanodavirus

Viral nervous necrosis (VNN) and viral encephalopathy and retinopathy (VER) have emerged as major constraints on culture of larval and juvenile and sometimes older marine fish worldwide leading to behavioural abnormalities and high mortalities [2,7]. The aetiological agents responsible for these diseases are small icosahedral viruses belonging to the *Betanodavirus *genus, within the *Nodaviridae *family. *Betanodavirus *genus is further divided in four species based on similarities in the variable region of the RNA2 and designated as Barfin flounder nervous necrosis virus (BFNNV), Redspotted grouper nervous necrosis virus (RGNNV), Striped jack nervous necrosis virus (SJNNV, the type species of the *Betanodavirus *genus) and Tiger puffer nervous necrosis virus (TPNNV) [8]. Black Beetle virus (BBV) and Flock House virus (FHV), the most studied viruses among the *Nodaviridae *family, infects insects and belongs to the second genus, *Alphanodavirus*. Fish nodaviruses are icosahedral, non-enveloped viruses with a commonly reported diameter of about 25 nm. The virus contains two single-stranded positive-sense RNA molecules, namely RNA1 (3.1 kb) and RNA2 (1.4 kb). Both RNA molecules are capped at their 5'-ends and lack poly(A) tails at their 3'-ends. The larger genomic segment, RNA1, encodes the RdRp (or protein A) and the smaller genomic segment, RNA2, encodes the coat protein. In addition, subgenomic RNA3, which is synthesized from RNA1 during RNA replication and not packaged into virions, encodes the two non-structural proteins B1 and B2.

The first recovery from cDNA of a virus belonging to the *Nodaviridae *family was reported by Kaesberg's group for BBV [33]. Because of the lack of an appropriate cell culture system, the establishment of such reverse genetics system for betanodaviruses was hampered. The first system was reported by Iwamoto and colleagues for SJNNV in 2001 [21]. This system was similar to that previously described for BBV and IPNV. It was based on the transfection into fish cells (E-11 cells cloned from striped snakehead SSN-1 cells) of synthetic RNA molecules of viral RNA 1 and 2 transcribed in vitro from linearized full-length cDNA plasmids with the T7RNAP and in the presence of a synthetic cap analogue (Figure 1). This system was further simplified by the use of a stable mammalian cell line expressing constitutively the T7RNAP, the BSR T7/5 cells [34]. In that goal, the cDNA plasmids encoding the RNA 1 and 2 were slightly modified by the fusion of a self-cleaving ribozyme δ at the 3'-end of each genome cDNA copy. Because of the temperature requirement of the mammalian cells and the betanodaviruses, 37°C versus 20-30°C, respectively, transfected-cells were incubated at 28°C for 48 h and then the cell lysates were inoculated on permissive fish cells at the optimal temperature for the viral replication [35,36]. Reverse genetics systems are now available for several betanodavirus species [21,35-37].

### Novirhabdovirus

IHNV and VHSV are causative agents of acute systemic diseases leading to high mortality, mainly, but not exclusively, in young cultured and wild salmonid fish all over the world and are listed as notifiable by the World Organisation for Animal Health (OIE) [2,38]. IHNV and VHSV are both enveloped bullet-shaped viruses (about 180 nm long and 70 nm in diameter) with a genome consisting of a non-segmented negative-sense single-stranded RNA molecule of about 11 kb [3,4]. These viruses belong to the *Rhabdoviridae *family within the *Mononegavirales *order. The most extensively studied mammalian rhabdoviruses are the rabies virus (RV) that belongs to the *Lyssavirus *genus and the vesicular stomatitis virus (VSV) that belongs to the *Vesiculovirus *genus. Similar to the mammalian rhabdoviruses, the IHNV and VHSV genomic RNA which encodes five structural proteins, is tightly encapsidated with a nucleoprotein (N), a polymerase-associated phosphoprotein (P) and the large RdRp (L) to form the helical ribonucleoprotein complex (RNP). The two other structural proteins are the matrix protein (M), which interacts with the RNP and the viral envelope and is involved in the budding step, and the unique viral surface glycoprotein (G) which is implicated in the entry step. In contrast to other rhabdoviruses, IHNV and VHSV genomes encode a small non structural NV (Non Virion) protein. Due to the presence of the NV gene, localized between the G and L genes, IHNV and VHSV are separated from the other rhabdoviruses and belong to the *Novirhabdovirus *genus [4].

The recovery of rhabdoviruses from cDNA presents a unique challenge because neither their genomic RNA nor their antigenomic complements serve as mRNA, and thus cannot be used directly to recover infectious viruses. In other words, the RNA of negative-stranded viruses (NSV) are not infectious upon transfection into a permissive cell line in contrast to those of positive-stranded viruses. The minimal infectious unit of NSV is the RNP complex which is able to drive both the sequential transcription of each viral gene and the replication of the full-length RNA genome. Based on this observation, the feasibility of producing infectious NSV entirely from cDNA was demonstrated by Conzelmann's group for RV, 15 years ago [14]. The goal of this system is to reconstruct into permissive cells a functional RNP complex. In that goal, cells are co-transfected with expression plasmids encoding N, P and L proteins together with a plasmid encoding the full-length viral antigenome, all under the control of the T7RNAP, which is provided to the cells by an infection with a recombinant vaccinia virus (vTF7-3; [26]) (Figure 1). In the co-transfected cells, the antigenome RNA is encapsidated by the N protein to form a suitable template for the RdRp (L and P) to drive the replication and the synthesis of genomic RNP. Once genomic RNP are available, an infectious cycle can be initiated with the expression of all viral proteins followed by the production of new infectious virions. Two tricks have contributed to the success of this system. The first one was the expression of an antigenome instead of a genome to prevent any hybridization with the mRNA expressed from the helper plasmids (N, P and L), which could prevent the initial encapsidation of the genome into a functional RNP complex and/or induce the host innate response mediated by the type I interferon. The second trick was the use of a self-cleaving ribozyme δ sequence fused to 3'-end of the antigenome cDNA allowing the expression of an antigenome RNA molecule having a precise 3'-end without any additional nucleotides, which were found deleterious for the rescue.

This system was then adapted to numerous viruses belonging to the *Mononegavirales *order [39] and was the starting point of various genetic manipulations (for review: [40,41]). Concerning the fish NSV, the description of a similar system, including plasmid design and T7RNAP supply by a recombinant vaccinia virus, was first given by Leong's group for the snakehead rhabdovirus (SHRV), a Novirhabdovirus which replicates at an elevated temperature of 31°C (Figure 1) [42]. This system was further adapted to low-temperature growing Novirhabdoviruses, such as IHNV and VHSV for which the optimal temperature of replication is 14°C [17,18,27]. This low-temperature requirement made the development of IHNV and VHSV reverse genetics more complex since vaccinia virus needs a temperature closed to 37°C to be able to express the T7RNAP. This obstacle was overcome by the selection of an IHNV and VHSV permissive cell line (fathead minnow EPC cells) able to undergo an incubation at 37°C for at least seven hours, a minimal incubation time required to express enough T7RNAP into the cells to drive the expression of each plasmid DNA before shifting the cells at 14°C (for review: [43]). Two kinds of vaccinia-virus-free reverse genetics systems for Novirhabdoviruses were then described removing the biosafety requirement imposed by the vaccinia virus manipulation and its highly recombinogenic property leading to nucleotide exchange between the transfected plasmids which can make difficult the recovery of certain mutants [18,44] (Figure 1). Leong's group described the SHRV recovery from a fish cell line constitutively expressing the T7RNAP (EPC-T7, derived from EPC cells) [45] and Vakharia's group demonstrated the recovery of both IHNV and VHSV using the cellular RNA polymerase II [15,16]. In this latter system, the cDNA encoding the IHNV N, P and L proteins and the viral antigenome were cloned under the control of a CMV promoter instead of that for the T7RNAP. In addition, a hammerhead ribozyme was fused to the 5'-end of the antigenome cDNA allowing the expression by the RNA polymerase II of an antigenome RNA molecule displaying a precise 5'-end.

## A wide panel of applications and breakthroughs

Reverse genetics makes it possible to manipulate viral RNA genomes through its cDNA copy and thus to evaluate genomic changes, such as gene deletion, heterologous gene exchange or addition and nucleotide substitution, on the biology and the pathogenesis of these recombinant viruses. All these reverse genetics-based studies over the last twelve years have provided new insights on fish RNA viruses that are presented above.

## Analysis of viral proteins of unknown function

### Novirhabdovirus NV protein

The ability to generate recombinant viruses by reverse genetics allows the opportunity to examine the role of the non-structural proteins in virus biology and pathogenic mechanisms. For instance, the NV proteins (111 to 122 amino acids), expressed by IHNV and VHSV in infected cells [46], were shown dispensable but necessary for an efficient virus replication in cell culture [15,17,18] and essential for virus pathogenicity in rainbow trout [47,48] and yellow perch (*Perca flavescens*) [15]. Indeed, both rVHSV-ΔNV and rIHNV-ΔNV, in which the entire gene was deleted, were highly reduced compared to their wild-type parents with regard to the efficiency of multicycle replication in cell culture, with up to 10 000-fold reduction. Moreover, rIHNV-ΔNV induced no mortality when administrated to rainbow trout by bath immersion and as low as 25% of cumulative mortality when intraperitonealy injected compared to the wild-type rIHNV which induced roughly 100% of cumulative mortality by both routes. Trans-complementation experiments using fish cells constitutively expressing NV protein clearly demonstrated that the lack of NV expression by itself was responsible for rIHNV-ΔNV phenotype, rather than a role of structural "spacer" for the NV gene in the IHNV genome [48]. The observation that the replacement of the NV gene by a reporter gene such as the chloramphenicol acetyltransferase (CAT) had no positive effect on rIHNV-ΔNV phenotype reinforced the above conclusion. Moreover, although the IHNV and VHSV NV proteins exhibit a low percentage of homology (31%) and no obvious conserved domains, both NV proteins shared a similar function since a recombinant IHNV virus, rIHNV-NV_VHSV_, in which the IHNV NV ORF was replaced by that of VHSV, was shown to replicate efficiently in fish cell and to induce similar cumulative percentage of mortality in vivo in trout compared to the wild-type IHNV [48]. Thus, altogether these results demonstrate the essential role of the NV protein of cold-water Novirhabdoviruses. In contrast to these findings, Leong's group, using a reverse genetics system established on the SHRV, a warm-water fish Novirhabdovirus, have observed no apparent role of the NV protein in replication in vitro as well as in fish pathogenesis [42,45]. This difference in NV requirement observed between IHNV and VHSV versus SHRV might be linked to the cold-temperature adaptation of IHNV and VHSV since no NV gene is found in warm-temperature rhaddoviruses such as the spring viremia of carp virus (SVCV, a fish vesiculovirus) and mammalian rhabdoviruses. But, it should be noted that the pathogenicity of the SHRV-ΔNV was assayed on zebrafish and by intraperitoneal injection which do not reflect the SHRV natural host (snakehead fish) and its presumed route of infection (waterborne). The intriguing result was that the elimination of the NV ORF by deletion or interruption with a premature stop codon had no effect on SHRV phenotype, whereas the deletion of the NV ORF together with the upstream gene stop/gene start signal has a deleterious effect. The authors speculate that the NV gene might have a spacing function regulating the SHRV genome transcription excluding any biological role of the NV protein which differs from the observations on both IHNV and VHSV viruses. It is well known that RNA viruses sustain mutations at a frequency of approximately of 10^-4 ^mutations per nucleotide copied, which is almost equivalent to one mutation per rhabdovirus genome copy, and thus exist as quasispecies [49]. This provides the capacity for the rapid outgrowth of variants that acquired a selective advantage. It can reasonably be assumed that an ORF without any critical function for the virus replication would accumulate several mutations leading to its rapid loss. Therefore, the fact that the NV ORF is still conserved in the SHRV genome and more generally in the *Novirhabdovirus *genus indicates that the NV protein by itself has a function. It could be of interest to replace the VHSV and IHNV NV gene by that of SHRV (24% and 20% of homology, respectively) to evaluate the potential of complementation of this NV protein on both virus phenotypes, and thus evaluate whether these three NV proteins that share less than 30% of homology have a common function.

### Betanodavirus B1 and B2 proteins

In *Alphanodavirus *genus, a subgenomic RNA3, corresponding to the 3'-end of RNA1, is synthesized by the viral RdRp during the replication. This subgenomic RNA3 encodes two small non-structural proteins, B1 and B2 (for review [50]). B2 protein is required for suppression of RNA silencing in infected insects, an adaptive antiviral response that controls viral infection in plants and animals. Efficient replication of FHV RNA and formation of progeny virus particles are critically dependent on the expression of B2 protein in vitro and in vivo [51-53]. The function of alphanodavirus B1 protein, which represents the C-terminus of RdRp protein, is currently unknown. Concerning the *Betanodavirus *genus, all viruses studied to date express a small RNA3 (about 0.4 kb) during their replication in infected cells and fish [54-56]. The RNA3 encodes a highly-conserved B2 protein among the betanodaviruses that consists of only 75 amino acids compared to their alphanodavirus counterparts which are 90 to 137 amino acids in size. Moreover, betanodavirus B2 protein does not possess any characteristic sequence motifs and domains in common with their alphanodavirus counterparts. Nonetheless, the B2 protein of betanodaviruses displays cellular RNA interference (RNAi) antagonist activity. For example, SJNNV B2 suppresses RNA silencing in a plant model [57], and greasy grouper nervous necrosis virus (GGNNV) B2 is important for the intracellular accumulation of viral RNA1 in a variety of cell types as shown with a RNA1-ΔB2 replicon vector, presumably due to its ability to block RNAi in animal cells [35]. Fenner and colleagues were unable to recover an infectious GGNNV-ΔB2 virus mutant using their reverse genetics system [35]. Indeed, although virus replication could be detected by indirect immunofluorescence assay after five consecutive passages on permissive cells, the genome analysis of these recovered viruses demonstrated that all of them had a C-to-T reversion at the B2 start codon restoring its expression. These results underline that betanodavirus B2 protein has an essential function at least during the virus replication. Additional data showed that GGNNV B2 binds long double-stranded RNA (dsRNA) preventing their recognition by the siRNA machinery and thus blocking the subsequent production of virus-derived small interfering RNA [58,59]. In contrast, B2 protein is unable to antagonize the dsRNA-mediated interferon response, as monitored by the *Mx *gene induction which is proportional to RNA1 accumulation in fish cells. Thus it seems that for betanodaviruses, which cause high mortalities in larval and juvenile marine fish, the most important host response at these fish developmental stages is RNA silencing [60]. To further complicate the understanding of B2 involvement in betanodavirus replication, recent studies indicate that B2 may play a dual role during the viral infection by first modulating the host siRNA response and then inducing mitochondria-mediated necrotic cell death [61]. The existence of a B1 ORF encoded by the betanodavirus subgenomic RNA3 is controversial. Although several groups reported that the B1 ORF may be absent in RNA3 [56,57], Chen and colleagues recently detected the early expression of B1 protein in RGNNV-infected fish cells using a B1 polyclonal antibody and showed that B1 may have an antagonist effect on B2 function by enhancing host cell viability [62]. Recovery of a recombinant virus in which the B1 ORF will be ablated (RGNNV-ΔB1) and observation of an enhancement of cell death in RGNNV-ΔB1-infected cells could give an additional argument to this new B1 function.

### Aquabirnavirus VP5 protein

The reverse genetics has also been used to investigate the potential role of VP5 on IPNV replication and virulence. Indeed, studies on recombinant IBDV lacking expression of VP5 showed that VP5 was nonessential for productive virus replication in cell culture, but played a key role in pathogenicity of IBDV [63]. Several recombinant IPNV, encoding for different size variants of VP5 or ablated for VP5 expression (IPNV-ΔVP5), were then successfully recovered. These virus mutants demonstrated that VP5 protein is not required for efficient virus growth in vitro, as previously observed for IBDV, but also in vivo in Atlantic salmon fry and post-smolt which differs from IBDV [64,65]. IPNV-ΔVP5 mutant was able to cause IPN disease in challenged Atlantic salmon post-smolts, leading to final cumulative mortality of 86% compared to 81% recorded with the wild-type virus [64]. Moreover, VP5 does not seem to be involved in the establishment of a carrier state since surviving salmon fry infected by IPNV-ΔVP5 were all IPNV carrier at least five months after being challenged as observed with those infected with the wild-type virus [64]. Altogether, these results strongly suggest that VP5 has no function as a virulence factor, but it can reasonably be assumed that VP5 provides a selective advantage since the majority of IPNV strains isolated to date encode the protein. It was previously reported that IPNV VP5, from the E1S strain, contains Bcl-2 homologous domains and that its stable expression in salmon cells enhances cell viability following exposure to apoptotic inducers [66]. However, this function is not conserved among IPNV strains. Indeed, IPNV serotype Sp strain and its VP5-deletion mutant derivative did not show any anti-apoptotic effect of VP5 expression in infected cell culture and in fish [67]. Finally, VP5 could be involved in the inhibition of interferon-stimulated gene expression [68]. But, based on the fact that inhibition of the host innate immune defence by a virus is required to establish a productive infection, the role of VP5 in the blockage of the interferon signalling does not appear to be crucial since IPNV-ΔVP5 was as virulent as its wild-type parent [64].

## Virus vectors

With efficient reverse genetics systems available for several major fish pathogens, the degree of flexibility of the genome of these viruses to genetic engineering and their ability to express foreign genes of interest were verified by several groups. For this purpose, negative-stranded viruses (NSV), such as rhabdoviruses, appear to be good candidates as vectors for transient gene expression compared to positive-stranded viruses (PSV) for many reasons (for review on mammalian virus-derived vectors [24,40,41]). Indeed, NSV genomes present a modular organization with individual cistrons defined by conserved gene start and gene end signals recognized by the viral RdRp and leading to the transcription of separate mRNA. In contrast, PSV genomes are expressed from unique polycistron mRNA which are translated into polyprotein precursors that follow a subsequent maturation by cellular or viral proteases. Moreover, the helical structure of the RNP complex of NSV brings two additional interesting features. First, due to the helical structure of the RNP, there is no defined size limit for the genomes in contrast to viruses with icosahedral capsids such as aquabirnaviruses, fish alphaviruses and betanodaviruses, in which the genome size is constrained. Nevertheless, although no size limit for NSV genome has been clearly defined, this rise of the genome size is always correlated with an increasing attenuation of the NSV vector. Second, the tight encapsidation of the NSV genome in the RNP reduces the chance of recombination as observed at high frequency with PSV, and prevents access of reverse transcriptases to the RNA avoiding any risk of cellular transformation. Finally, due to a decreasing gradient of gene expression along the NSV genome from the 3'-end to the 5'-end (i.e. the expression of the first gene is the most abundant and the last gene the least); the level of expression of the gene of interest can be modulated by moving its position in the gene order. Thus, NSV modular organization can be readily manipulated for the insertion and stable maintenance of nonessential sequences such as foreign genes. In both cases, the host restriction has to be considered for a wider use of the NSV and PSV vectors to other fish species.

### Antigenic chimeric viruses: example of Novirhabdovirus glycoprotein gene exchange

An attractive method to express foreign antigens by a recombinant virus is to replace its major protective surface antigen with that of another virus of interest. Such constructs have been generated by exchanging the unique surface glycoprotein of different distantly-related and antigenically-distinct fish rhabdoviruses [45,69]. The IHNV genome was engineered such that the IHNV G gene was replaced by those of VHSV (another fish *Novirhabdovirus*, 51% homology between both proteins) and the spring viremia of carp virus (SVCV; a fish vesiculovirus, 29% homology between both G) [69]. Both chimeric recombinant viruses, IHNV-Gvhsv and IHNV-Gsvcv, were successfully recovered and were found as efficient as IHNV for multicycle replication in cell culture. The only remarkable feature was the plaque morphology induced by IHNV-Gvhsv which was of intermediate size compared to those induced by both parental viruses (IHNV, small plaques and VHSV, large plaques). However, although IHNV-Gvhsv was as virulent as its biological parents (IHNV and VHSV) in rainbow trout, the histological lesions induced by this recombinant virus in trout were more similar to those obtained with IHNV [70,71]. Interestingly, although SVCV is not pathogenic in rainbow trout, a high cumulative mortality could be observed with IHNV-Gsvcv by bath immersion (93% of cumulative mortality with IHNV-Gsvcv versus 95% with IHNV), demonstrating that SVCV G is fully functional in the IHNV background and that another SVCV protein should be involved in the host restriction [70]. The efficient recovery of these chimeric viruses could be explained by the effective incorporation of the foreign glycoprotein into the viral particles as demonstrated on sucrose-gradient purified IHNV-Gvhsv and IHNV-Gsvcv virions. This observation shows that IHNV does not require any specific IHNV G-derived sequence to efficiently incorporate a heterologous glycoprotein. This is similar to previous observations with the mammalian rhabdovirus VSV for which a correct length of the cytoplasmic tail is more important than specific amino acid sequences (for review [41]). In contrast, efficient incorporation of foreign glycoprotein into RV virions requires that the cytoplasmic tail of the RV glycoprotein is conserved. The IHNV flexibility for heterologous glycoprotein incorporation was further demonstrated by the IHNV G exchange with the mammalian VSV counterpart [70]. The recombinant IHNV-Gvsv was fully efficient for replication in fish cells at low temperature (14°C). A similar observation was performed with a recombinant SHRV, a warm-water fish Novirhabdovirus, in which the SHRV glycoprotein was exchange by that of IHNV (SHRV-Gihnv; 52% of homology between both G) [45]. Finally, the high flexibility of IHNV was further demonstrated by simultaneous exchange of IHNV G and M genes with those of VHSV [69]. In addition, these recombinant viruses were useful to investigate whether the glycoprotein was the only protein responsible for the temperature growth restriction of IHNV. Indeed, IHNV and VHSV cannot grow at a temperature above 20°C, whereas SVCV, SHRV and VSV replicate well up to 28°C, 30°C and 37°C, respectively. However, SHRV-Gihnv only gives limited replication at 30°C pointing out the conformational instability of the IHNV glycoprotein at this temperature [45]. Moreover, IHNV-Gsvcv and IHNV-Gvsv do not replicate at temperatures higher than 20°C, demonstrating that the G is not the only protein involved in the temperature growth restriction and that probably the polymerase complex requires an optimal temperature for its efficient activity [69,70].

### Insertion of a foreign gene

As explained above (see section 3.2), the strategy used to insert an additional ORF in RNA viruses depends on the genome organization. Concerning NSV, such as rhabdoviruses, the sequential transcription is initiated by the RdRp upstream of each ORF by the recognition of a gene start signal and ended at a gene end signal that serve as transcription stop and polyadenylation signals (for review on mammalian NSV [40,41]). A functional cassette expressing a foreign gene has to be engineered as a cDNA including the desired ORF flanked by the viral gene start and gene end signals. These signals can be added artificially or recovered from an existing gene by exchanging the viral ORF by that of the gene of interest or by engineering a fusion protein. In the case of an artificial cassette, the cDNA is then inserted into a non-coding intergenic region found between each viral gene. Due to the gradient of expression in NSV genome, the insertion site into the viral gene order will influence the level of expression of the foreign gene as well as the level of attenuation of the virus vector. Indeed, the insertion of a foreign sequence decreases the expression of the following genes and has more or less effect on the replication of the recombinant virus.

Both strategies were tested for IHNV and VHSV [15-17,47,72]. First, the IHNV NV ORF was replaced by different reporter genes as CAT, the green fluorescent protein (GFP), the *Renilla *luciferase (LUC), the VHSV G and different other virus-antigen encoding genes ([18,47,73,74], Harmache A. and Brémont M., unpublished data). Although these viruses were highly attenuated by the deletion of the NV gene (see section 3.1), they were all able to efficiently express detectable amount of the foreign protein in cell culture. In vivo, these viruses were non-pathogenic in rainbow trout and too much attenuated to be used as vaccine vectors (see below) [47,48,74]. Second, artificial cassettes of expression were then constructed for both IHNV and VHSV [15-17,47,72]. IHNV and VHSV share similar consensus sequences for the gene end and gene start signals except for the hairpin loop structure shown to be part of the end of the IHNV gene start signal: (UCURUC(U)_7_RCCGUG(N)_4_CACR versus UCUAUC(U)_7_RCCGUG, respectively). These signals were successfully used to express a large panel of reporter genes: LUC, GFP and red fluorescent proteins such as Cherry and Tomato [15-17,47] and viral antigens from ISAV, IPNV and SDV ([72], Harmache A. and Brémont M., unpublished data). All these recombinant viruses were readily recovered at high titer demonstrating that the insertion of an additional gene in the intergenic region between P-M genes (IHNV), M-G genes (IHNV and VHSV) and N-P genes (VHSV) have a limited effect on the efficacy of replication at least in cell culture. Moreover, recombinant IHNV viruses with insertion between M-G genes were shown fully pathogenic in rainbow trout by bath immersion inducing final cumulative mortalities similar to that induced by IHNV [47]. As compared to other RNA viruses, foreign sequence introduced into IHNV and VHSV genomes were surprisingly stable after multiple passages in cell culture (up to 20 passages) and in rainbow trout (Biacchesi S. and Brémont M., unpublished data). For a mammalian rhabdovirus (RV), it was shown that the expression of a reporter gene that does not affect the virus replication was stable over 25 passages in cell culture [75].

These recombinant viruses, expressing a foreign gene, were used for several applications that led to amazing observations. The most interesting example was achieved with a recombinant IHNV expressing the LUC gene and a non-invasive bioluminescence imaging device [47]. Indeed, this technology provided evidence that the fins were the portal entry of IHNV into the rainbow trout. The authors also showed that a non-pathogenic IHNV-ΔNV, in which the NV gene was replaced by the LUC gene, had a limited replication exclusively in the fins and could persist in fins up to 3 weeks postinfection without any further propagation into the infected fish. These observations explained why IHNV-ΔNV could not be detected by RT-PCR in internal organs and in part the absence of induced-antibody expression [48,74]. Although IHNV-ΔNV-immunized fish were fully protected against a subsequent challenge with a highly-pathogenic IHNV strain at 30 days post-immunization [48], mortalities could be recorded for challenge performed at a later period post-immunization (Harmache A. and Brémont M., unpublished data). Altogether, the results suggest that the early protection was the consequence of an interference phenomenon in the fins between the IHNV-ΔNV and the wild-type virus.

Recombinant VHSV and IHNV, expressing Tomato and GFP reporter gene, respectively, were simultaneously used to coinfect fish cell monolayers [17]. It was observed that up to 74% of the cell monolayer could be coinfected by both viruses demonstrating limited superinfection exclusion between these two Novirhabdoviruses. These results suggest that fish living in endemic region for IHNV and VHSV are likely to be coinfected by both viruses, as previously observed during experimental coinfection [76]. As mentioned above (in section 3.2.1), IHNV has an extreme flexibility to accommodate heterologous structural proteins, such as foreign glycoproteins. So, it could be assumed that during coinfection such events occur and lead to chimeric and/or pseudotyped viruses with unique phenotype in fish. In contrast, it is less probable that recombination occurs between both viruses since IHNV and VHSV display a high genetic divergence (57% of position identity between both genomes) and recombination is extremely rare in NSV [77,78].

Finally, Harmache et al. investigated the potential of IHNV as vaccine vehicle against other fish viral diseases ([72] and Harmache A. and Brémont M., unpublished data). They reported the production of recombinant IHNV based vaccine vectors that carry three expression cassettes of foreign antigens derived from four of the most devastating fish viral pathogens: VHSV G glycoprotein, IPNV VP2, SDV C-E3-E2 and infectious salmon anaemia virus (ISAV) hemagglutinin-esterase glycoprotein and fusion protein. They found that the three foreign antigens were efficiently and stably expressed by the recombinant IHNV vector although its genome was increased in size more than 50% compared to the wild-type genome (about 5.5 kb of foreign sequences). Because of this important increase of the genome size together with the effect on the expression of the downstream viral genes, these IHNV vectors were unsurprisingly highly attenuated in trout leading to only 10% of cumulative mortality. Nevertheless, surviving trout were protected against subsequent challenges with not only the pathogenic IHNV but also with the targeted viruses. Thus, genome size increase has an effective attenuation effect which can be assumed relatively stable due to the low rate of recombination observed in NSV genomes [77,78]. However, it cannot be totally excluded that the foreign gene sequences by themselves or their products are somewhat responsible for the observed attenuation.

The use of fish PSV as gene vector has been less explored compared to fish NSV. Indeed, the flexibility of the PSV genomes for genetic engineering and their ability to stably express foreign genes of interest are impaired for several reasons. PSV genomes are expressed as polyprotein precursors that require a subsequent maturation by cellular or viral proteases. Moreover, because of packaging constraints (icosahedral capsids), large inserts (> 2 kb) are not stable upon passaging and rapidly removed by recombination which is observed at high frequency with PSV (for review on mammalian alphavirus [24]). Therefore, a foreign gene has to be engineered as a cDNA including the desired ORF flanked by a protease cleavage site and inserted in the genome in frame with the viral ORF without ablating a potential overlapping ORF. The gene or the antigen of interest can also be expressed as a fusion protein. These kinds of genome manipulation are generally lethal for the vector and thus various insertion sites have to be tested in the goal to generate an infectious virus. Concerning the alphaviruses, the identification of the subgenomic RNA promoter element allowed the construction of viral genomes with additional subgenomic RNA promoters leading to the synthesis of additional subgenomic mRNA. This strategy was applied to SDV by Moriette and colleagues [19]. The SDV replication was not drastically impaired when an extra gene (GFP or LUC) was present in the viral genome. The second subgenomic mRNA was inserted either upstream or downstream to the structural protein genes and up to three additional subgenomic mRNAs were tested. These data demonstrated that SDV is able to encapsidate a genome that could be more than 20% longer than that of the wild-type virus. However, expression of GFP was highly unstable and no longer detected after one additional passage of the recombinant virus in cell culture. However, the long-term success of developing mammalian alphavirus as vaccine vector should not be underestimated. Further development should be necessary to increase the stability of the foreign gene such as expression of the foreign protein as a cleavable component of the viral structural polyprotein or insertion of the foreign protein in a viral nonstructural protein as described for SINV [79,80]. No example of betanodavirus and aquabirnavirus used as gene vector are available in the literature underlying the difficulty to generate gene vector**s **with PSV.

## Virulence and host specificity factors

The reverse genetics is a powerful tool to map in the virus genome virulence and host specificity factors based on previous putative nucleotide changes determined after genome sequence comparisons of virus strains displaying different phenotypes. For instance, amino acid sequence comparisons of IPNV VP2 protein from various field isolates belonging to the Sp serotype, which exhibit different mortality rates in Atlantic salmon fry, identified putative residues responsible for modulating virulence between strains [81,82]. The significance of these amino acid changes in VP2 were later confirmed with recombinant reassortant and chimeric viruses generated by reverse genetics. Indeed, Song et al. demonstrated that virulence and also adaptation to cell culture are controlled by two VP2 amino acids at positions 217 and 221 [83]. Highly virulent strains encode Thr217 and Ala221, while moderate to low virulent strains have Pro217 and Ala221 and variants having Thr221 are almost avirulent, irrespective of the amino acid in position 217. In parallel, these observations allowed better understanding of the molecular basis of the cell culture attenuation by detecting the Thr221 substitution in VP2 after 10 passages of a virulent strain in a certain fish cell line. This virus became highly attenuated and induced only 15% of cumulative mortality in Atlantic salmon fry, compared to 68% mortality recorded with its virulent parent strain. The recent publication of the crystal structure of IPNV virion demonstrated that these two amino acid residues at position 217 and 221 are localized in the most peripheral loop at the top of the VP2 spike [30]. Since these two residues are exposed and do not participate in VP2 folding and interactions between subunits, their major role in IPNV strain virulence suggest that they may be involved in attachment to the target cell.

Similar approaches were undertaken to study betanodavirus host specificity and temperature restriction. Although a high overall percentage of homology is found between the four types of betanodavirus isolates, some isolates have marked host specificity. For example, SJNNV causes disease only in the striped jack (*Pseudocaranx dentex*), whereas members of RGNNV type, such as sevenband grouper nervous necrosis virus (SGNNV), has a broad host range and causes diseases for a variety of warm-water fish species, particularly for sevenband grouper (*Epinephelus septemfasciatus*). Recombinant reassortant and chimeric viruses were generated by using reverse genetics systems established for SJNNV, RGNNV and SGNNV [37,84]. The authors of these two reports showed that the variable region of the RNA2, which encodes the C-terminal region of the coat protein, controls host specificity in betanodaviruses. Indeed, RNA2 chimeric viruses from SJNNV and RGNNV demonstrated that SJNNV mutants containing the variable region of RGNNV RNA2 infected sevenband grouper larvae as efficiently as RGNNV, while RGNNV mutants containing the variable region of SJNNV RNA2 infected striped jack larvae similarly to SJNNV. Since the C-terminal of the coat protein was shown by electron cryomicroscopy to form the surface-protruding domain of virus-like particles [85], its major role in betanodavirus host specificity suggests that this region may be involved in attachment to the target cell. These two reverse genetics systems were further used to investigate the temperature restriction observed between SJNNV and RGNNV [86]. Whereas the optimal temperature for the growth of SJNNV is 20-25°C, that of RGNNV is 25-30°C. Thus, a set of recombinant reassortant and chimeric viruses from SJNNV and RGNNV were recovered and their optimal temperature for replication in cell culture was determined. Although both RNA1 and RNA2 seem to control this temperature sensitivity as shown by reassortant viruses, chimeric viruses indicate that the RGNNV RNA1 region, encoding amino acid residues 1 to 445 of the RdRp was implicated in this restriction. Interestingly, this region encodes a mitochondrial-targeting signal and is located outside of the catalytic domain of the RdRp.

For alphaviruses, incidental mutations introduced during the RT-PCR steps needed to construct infectious cDNA for SDV have also contributed to identify potential attenuating position in their genomes. Concerning SDV, several nucleotide substitutions have been mapped in the structural and non-structural genes of the infectious cDNA compared to the published sequence (see details in [19,25]). Although the recombinant SDV (rSDV) was fully efficient for replication in cell culture reaching a final titer of 3 × 10^8 ^PFU/mL similar to the field isolate, the virus was totally attenuated in rainbow trout when administrated by either bath immersion or injection. Nevertheless, high titers (about 10^8 ^PFU/mL of serum) of rSDV could be harvested from sacrificed fish up to three weeks post-infection. Immunized rainbow trout acquired long-lasting (at least seven months) and complete protection against challenges with a highly virulent SDV strain by bath immersion or injection. In addition, rSDV-vaccinated fish were also protected from a challenge with the wild-type SPDV, a closely related but distinct salmonid alphavirus. It has to be mentioned that in addition to the genome sequence mutations, the temperature of virus adaptation to the cell culture seems to play a role in the SDV attenuation. Indeed, naïve trout infected with the wild-type SDV adapted to 14°C (SDV14) or with that adapted to 10°C (SDV10) exhibited different cumulative mortality rates two months postinfection, roughly 80% and 8%, respectively. The rSDV was also recovered at 10°C which could explain in part its in vivo attenuated phenotype. The effect of the temperature at which the viruses were produced on the virulence was established by adapting rSDV and SDV10 to grow at 14°C. Indeed, the resulting viruses induced higher cumulative mortality rates in trout: from 0 to 24% for rSDV and 7% to 30% for SDV10, respectively. The comparison of the nucleotide sequences encoding the structural proteins pinpointed six major amino acid changes (three in E2, two in 6K and one in E1) between rSDV and its 14°C-adapted derivative. Moreover, as SDV10 was derived from SDV14 after plaque purification, it could be assumed that SDV14 inoculum represents a quasispecies population pathogenic for trout and that the viral heterogeneity was greatly reduced for SDV10 and even more for the rSDV derived from a single cDNA clone. This hypothesis was in part demonstrated by the partial sequencing of the three virus genomes which pinpointed a large number of amino acid changes, roughly 60 and 20 in the non-structural genes of SDV14 and SDV10 compared to the counterpart region in rSDV [25].

Concerning IHNV, the introduction of four incidental mutations during the construction of the full-length cDNA construct was enough to highly attenuate the recovered virus. This recombinant IHNV, containing 2 amino acid substitutions in the N, one in the P and one in the M, induced less than 10% of cumulative mortality in trout by bath immersion and was found to be a strong inducer of IHNV-specific antibodies [70,71]. Surprisingly, when two additional mutations, previously described in the G of a neutralization-escape IHNV mutant (RB1 strain) as highly attenuating (amino acids 78 and 218) [87], were introduced in this virus, virus-induced mortality was not decreased [71]. This observation together with the fact that a recombinant IHNV containing G78 and G218 as unique mutations was only slightly attenuated suggest that the RB1 strain, for which the G was the only gene fully sequenced, have other changes in its genome [71]. No example of putative virulence factor screening by reverse genetics has been reported for Novirhabdoviruses in the literature yet. However, recombinant IHNV viruses containing the G and/or NV genes from North American U or M IHNV strains have been constructed and tested for host-specific virulence in both rainbow trout and sockeye salmon (*Oncorhynchus nerka*) (Kurath G., Harmache A., and Brémont M., unpublished data). The U and M IHNV strains differ in host specific virulence: U strain is virulent in sockeye salmon but not rainbow trout, and the M strain is virulent in rainbow trout but not sockeye salmon. These studies revealed that the G and NV genes do not contain the determinants of host-specific virulence for these IHNV strains, suggesting that the molecular mechanism(s) of host-specificity in this case must be different from the viral coat protein host cell attachment mechanism described for IPNV and betanodaviruses. Finally, in recent decades, several VHSV and IHNV strains have been isolated and fully sequenced. For instance, several VHSV strains have been isolated from several marine fish species (for review see [38]). The interesting observation was that VHSV isolates originating from marine fish show low pathogenicity to rainbow trout and Atlantic salmon, although some of these isolates are pathogenic for turbot (*Scophthalmus maximus*). Several nearly complete genome sequences from marine and freshwater isolates displaying variable level of virulence in rainbow trout are now available in GenBank. From the analysis of these sequences, several groups have already pinpointed putative amino acid residues that could be involved in VHSV strain virulence [88,89]. Interestingly, these authors point out that as few as 4 to 10 amino acid changes were identically substituted between the marine and freshwater strains suggesting that only a limited number of amino acid residues might be involved in the level of virulence between isolates. Using the newly described reverse genetics system based on the hypervirulent freshwater VHSV 23-75 strain [17], these amino acid residues could be easily tested in the goal to determine their biological relevance in VHSV virulence.

## Conclusion remarks

Since the description of the first reverse genetics system for a fish RNA virus in 1998 [20], several other systems have been established for the major fish RNA virus pathogens. These technologies have largely contributed to a better understanding of the biology of these viruses in term of virulence and host specificity factors as well as virus-host interactions and virus entry in the host. They also allowed the genomic manipulation of these viruses and thus the generation of gene-deletion mutants, attenuated virus vaccines and gene vectors, such as multivalent live attenuated vaccines and tracer viruses expressing reporter genes. Live attenuated virus vaccines would be the optimal fish vaccines in term of cost, protective efficacy and ease of administration (bath immersion instead of injection for traditional vaccines). But, additional works have to be undertaken to remove any existing residual virulence and insure their stability avoiding any reversion to a wild-type phenotype in the goal to address the safety concerns of the consumers and to the environment (for review on development of safer live attenuated virus vaccines see [90]). Regulatory constraints are the main obstacle for this kind of vaccines especially in view of aquaculture practices where cultured fish live in ponds or nets with no physical barriers to wild stocks. Indeed, even if non-pathogenic for the targeted animal, the live virus vaccine has to be safe to all other species in the aquatic environment. One alternative would be the use of these live virus vaccines in a confined environment during the immunization protocol and then assure the absence of any persistent virus infection before housing the immunized fish in an open aquatic environment. Furthermore, reverse genetics system**s **could be used to generate single-replication cycle virus-based vaccine vectors such as alphavirus replicons. Although this kind of vaccine was found safe and protective in mammals after injection, the immunogenicity in fish of such vaccines should be demonstrated when administrated by bath immersion. The next several years will clearly be very exciting time in fish RNA virus research since advances in virus genome manipulation, fish genome sequencing and fish immune system characterization have greatly increased these last years.

## Competing interests

The authors declare that they have no competing interests.
